# Supplementary material for: Magnetic Clouds: Solar Cycle Dependence, Sources, and Geomagnetic Impacts
Source: Sol Phys. 2018 Oct 2;293(10):135. doi: 10.1007/s11207-018-1356-8 (PMC6190751; doi:10.1007/s11207-018-1356-8)
Supplement: Supplementary file 1 — (DOCX 105 kB) [file 11207_2018_1356_MOESM1_ESM.docx]

Magnetic Cloud List

———————————————————

year mth day hr mn V |B| Bs

km/s nT nT

———————————————————

1995 02 07 00 00 480 13 -10

1995 03 04 08 00 450 12 -10

1995 05 16 06 00 380 25 -15

1995 06 30 15 00 490 11 -7

1995 08 22 21 00 390 11 -8

1995 09 15 17 00 450 12 0

1995 09 27 12 00 400 14 -12

1995 10 18 20 00 405 22 -20

1996 05 27 17 30 420 11 -9

1996 07 01 20 00 360 14 -7

1996 08 07 09 00 360 08 -4

1996 12 24 05 00 390 12 -6

1997 01 10 03 00 500 15 -13

1997 02 10 03 00 460 09 -8

1997 04 11 06 00 470 22 -5

1997 04 21 12 00 400 14 -7

1997 05 15 10 30 450 25 -24

1997 05 26 13 00 350 10 -10

1997 07 15 08 00 370 12 -10

1997 08 03 10 00 480 17 -11

1997 09 03 13 00 410 18 -14

1997 09 18 06 00 320 12 -10

1997 09 22 01 00 480 18 0

1997 10 01 01 00 480 10 0

1997 10 10 20 00 430 14 -10

1997 11 07 07 00 470 18 -12

1997 11 22 13 00 520 19 -12

1997 12 10 18 00 390 16 -11

1997 12 30 09 00 360 14 -11

1998 01 07 00 00 410 20 -10

1998 01 21 06 00 460 18 0

1998 02 04 06 00 360 14 -8

1998 02 17 12 00 400 16 -12

1998 02 19 00 00 420 12 -5

1998 03 04 12 00 370 12 -7

1998 05 02 12 00 640 14 -13

1998 05 16 00 00 400 16 0

1998 06 02 11 00 410 12 -4

1998 06 14 05 00 350 12 -11

1998 06 25 00 00 480 17 -12

1998 07 11 16 00 380 14 -7

1998 08 20 08 00 340 16 -14

1998 08 27 06 00 700 16 -11

1998 09 25 08 00 820 19 -5

1998 10 19 04 00 410 26 -22

1998 11 08 03 00 650 36 -16

1998 11 13 05 00 400 20 -20

1999 02 18 16 00 650 14 -11

1999 02 28 13 30 380 17 -9

1999 04 16 22 00 460 25 -15

1999 04 21 05 00 550 10 -6

1999 06 03 01 00 400 12 0

1999 06 26 08 00 360 15 -8

1999 08 09 01 00 410 12 -7

1999 09 23 00 00 550 28 -8

1999 10 21 06 00 400 35 -26

2000 02 21 12 00 410 17 -5

2000 03 01 04 00 520 11 -7

2000 03 19 04 00 390 11 -7

2000 03 28 04 00 400 10 -4

2000 05 07 08 00 380 15 00

2000 05 12 00 00 300 10 -9

2000 06 08 18 00 750 17 -7

2000 06 26 00 00 550 16 -10

2000 07 01 09 00 400 10 00

2000 07 12 00 00 530 11 00

2000 07 15 20 00 1000 50 -45

2000 07 20 00 00 600 12 -7

2000 07 28 14 00 470 20 -6

2000 07 31 22 00 450 12 -6

2000 08 12 07 00 670 35 -25

2000 08 23 17 00 300 12 0

2000 09 18 00 00 820 37 0

2000 10 03 15 00 430 18 -11

2000 10 13 16 00 420 12 -12

2000 10 28 21 00 410 19 -18

2000 11 07 00 00 600 25 -10

2000 12 23 00 00 320 14 -12

2001 03 04 06 00 450 12 -10

2001 03 12 12 00 370 11 -3

2001 03 20 00 00 450 22 -20

2001 03 27 18 00 620 25 -18

2001 03 31 13 00 600 35 -32

2001 04 11 23 00 710 30 0

2001 04 22 00 00 380 16 -16

2001 04 28 15 00 700 20 -17

2001 05 07 19 00 370 08 -6

2001 05 28 11 00 500 10 -8

2001 06 19 00 00 400 16 0

2001 07 10 16 00 370 10 -7

2001 08 18 00 00 600 25 0

2001 09 14 01 00 400 17 0

2001 09 29 18 00 600 15 -7

2001 10 01 00 00 520 19 -10

2001 10 03 06 00 530 23 -20

2001 10 12 03 00 580 24 -7

2001 10 22 00 00 680 17 -11

2001 10 31 21 00 390 12 -14

2001 11 05 21 00 800 62 -42

2001 11 15 17 00 350 17 0

2001 11 24 20 00 800 20 0

2002 02 02 02 00 350 13 -12

2002 02 28 18 00 400 17 -11

2002 03 02 12 00 400 11 -5

2002 03 20 00 00 370 15 -7

2002 03 24 11 00 450 20 -7

2002 04 18 04 00 500 15 -12

2002 05 19 04 00 490 19 -10

2002 05 23 21 00 800 10 0

2002 08 02 07 00 510 13 -8

2002 08 20 17 00 500 12 -10

2002 09 03 18 00 380 18 0

2002 09 08 22 00 500 10 -6

2002 09 23 06 00 400 10 -4

2002 10 01 00 00 380 25 -12

2002 10 04 05 00 400 12 -11

2003 01 27 02 00 580 12 0

2003 03 20 12 00 680 12 -7

2003 05 30 03 00 630 30 0

2003 08 05 01 00 420 13 -10

2003 08 18 08 00 500 20 -15

2003 09 01 08 00 410 11 -5

2003 10 01 22 00 320 19 -10

2003 10 29 12 00 800 45 -18

2003 11 20 20 00 550 50 -45

2004 01 22 09 00 640 14 -5

2004 02 11 08 00 450 22 -12

2004 04 04 00 00 500 18 -2

2004 07 24 16 00 600 22 -20

2004 07 27 04 00 1000 25 -18

2004 08 29 20 00 400 13 -10

2004 11 07 23 00 720 46 -42

2004 11 09 21 00 800 40 -26

2004 11 12 08 00 600 12 -5

2004 12 05 08 00 450 30 -7

2004 12 13 00 00 400 16 -10

2005 01 07 17 00 520 22 -15

2005 01 22 00 00 820 27 0

2005 02 16 12 00 415 13 -7

2005 03 16 16 00 400 10 -5

2005 05 15 09 00 940 53 -10

2005 05 20 15 00 500 15 -10

2005 05 29 10 00 500 19 -16

2005 06 12 16 00 500 18 -15

2005 06 15 06 00 500 10 -6

2005 07 10 12 00 450 25 -14

2005 07 17 13 00 450 15 -9

2005 08 02 09 00 470 11 0

2005 08 24 16 00 710 25 -12

2005 08 31 08 00 400 19 -12

2005 09 11 06 00 980 18 -5

2005 10 31 02 00 400 13 -8

2005 12 31 16 00 470 16 -5

2006 01 25 08 00 400 11 -8

2006 02 05 23 00 370 12 -9

2006 04 04 07 00 320 10 -10

2006 04 13 16 00 500 20 -12

2006 06 06 11 00 460 12 -7

2006 08 20 00 30 410 13 0

2006 08 31 06 00 400 10 -6

2006 09 30 10 00 400 18 -6

2006 11 29 10 00 430 16 -8

2006 12 15 00 00 810 18 -15

2007 01 14 11 45 354 14 -7

2007 02 26 05 00 400 11 0

2007 03 24 06 00 380 12 -8

2007 05 22 01 00 400 13 -3

2007 06 08 06 00 300 09 -5

2007 11 20 00 00 450 19 -16

2008 03 08 16 00 390 16 -14

2008 09 03 16 00 450 13 -8

2008 11 07 06 00 360 11 -5

2008 12 04 12 00 400 08 -4

2008 12 17 06 00 340 10 -6

2009 01 26 06 00 390 10 -8

2009 02 04 00 00 390 11 -9

2009 03 03 12 00 380 11 -5

2009 03 12 01 00 350 18 -11

2009 06 28 00 00 400 10 -8

2009 07 21 03 00 350 08 -5

2009 08 05 10 00 370 12 -5

2009 08 30 08 00 400 13 -6

2009 09 30 08 00 350 10 -4

2009 10 22 12 00 350 11 -7

2009 10 29 06 00 350 12 -6

2009 11 14 06 00 330 08 -6

2009 12 12 23 30 300 08 -3

2010 02 08 00 00 400 11 0

2010 04 05 12 00 700 13 -7

2010 04 12 00 30 400 12 0

2010 05 18 09 00 350 08 -7

2010 05 28 23 00 390 15 -14

2010 08 04 10 00 550 13 -8

2010 09 15 01 00 380 10 -3

2010 10 11 09 00 350 14 -12

2010 10 17 01 00 350 10 -6

2010 10 31 07 00 380 11 0

2010 12 20 02 00 390 09 -7

2010 12 28 08 00 350 13 -11

2011 01 24 10 00 400 08 -4

2011 02 04 09 00 400 16 -10

2011 02 18 21 00 500 14 -5

2011 03 30 01 00 380 14 -7

2011 04 11 18 00 500 15 -8

2011 04 24 00 00 400 10 -5

2011 05 28 00 00 500 13 -10

2011 06 05 09 00 500 15 -5

2011 06 17 03 00 550 10 -5

2011 09 09 22 00 500 15 -7

2011 09 17 12 00 500 14 -8

2011 10 05 13 00 450 13 0

2011 10 25 00 00 525 24 -11

2011 10 31 18 00 400 12 -7

2011 11 04 23 00 290 08 -4

2011 11 29 20 00 425 10 -5

2011 12 29 23 00 400 09 0

2012 01 02 20 00 400 12 -7

2012 01 05 19 00 300 10 -5

2012 01 22 23 30 410 14 -10

2012 02 01 06 00 380 08 -5

2012 02 14 22 00 390 09 -8

2012 03 09 00 30 700 17 -15

2012 03 15 21 00 700 15 -10

2012 03 22 20 00 400 10 -7

2012 04 04 20 00 320 09 -8

2012 04 05 18 00 320 12 -6

2012 04 23 20 00 380 16 -14

2012 05 04 02 00 310 08 0

2012 05 16 21 00 370 12 -7

2012 05 22 04 00 400 13 -5

2012 06 01 03 00 360 10 0

2012 06 11 15 00 400 10 -10

2012 06 16 23 00 500 40 -15

2012 07 09 00 00 410 12 -11

2012 07 15 07 00 699 27 -17

2012 07 28 06 00 320 10 -8

2012 08 08 04 00 400 09 -6

2012 08 12 20 00 380 12 -5

2012 08 18 20 00 400 12 0

2012 09 06 02 00 400 11 -3

2012 09 30 23 00 400 21 -17

2012 10 08 18 00 400 17 -16

2012 10 12 18 00 480 13 -11

2012 11 01 00 30 360 15 -12

2012 11 13 08 00 380 21 -17

2012 11 24 12 00 400 15 0

2012 12 02 09 00 320 10 -2

2013 01 17 12 00 400 15 -10

2013 03 18 00 00 600 13 0

2013 04 15 01 00 510 11 0

2013 04 30 14 00 400 10 -8

2013 05 01 11 00 410 11 -7

2013 05 14 08 00 380 08 -5

2013 05 26 00 00 700 12 -4

2013 06 06 16 00 480 14 -13

2013 06 28 04 00 420 14 -12

2013 07 05 19 00 350 13 -11

2013 07 10 16 00 450 11 -5

2013 07 13 03 00 480 17 -10

2013 08 05 00 00 550 09 -6

2013 09 03 03 00 370 09 -5

2013 10 02 22 00 500 08 -5

2013 10 09 10 00 580 12 -3

2013 12 01 10 00 500 10 0

2013 12 15 16 00 470 10 -4

2013 12 25 06 00 300 11 -9

2014 02 08 08 00 450 10 -5

2014 02 16 02 30 400 17 -8

2014 02 19 15 00 520 15 -11

2014 02 23 06 00 470 12 -10

2014 02 28 23 00 470 16 -6

2014 03 14 02 00 500 09 -4

2014 04 06 00 00 370 18 -5

2014 04 11 06 00 370 10 -8

2014 04 29 22 00 320 10 -9

2014 05 23 02 00 500 13 -6

2014 05 30 12 00 350 11 -9

2014 06 17 04 00 350 10 -6

2014 06 29 23 00 360 09 -5

2014 07 13 12 00 350 11 0

2014 08 10 10 00 350 10 -5

2014 08 19 21 00 380 21 -9

2014 08 27 00 00 300 14 -10

2014 09 06 17 00 400 10 -5

2014 09 13 00 00 720 32 0

2014 11 04 00 00 400 12 -4

2014 12 06 04 00 450 22 0

2014 12 22 04 00 400 26 -11

2015 01 07 06 00 450 21 -17

2015 02 15 12 00 380 11 -6

2015 02 16 15 00 380 15 -3

2015 03 17 12 00 600 31 -22

2015 03 28 08 00 420 15 0

2015 03 31 18 00 400 10 0

2015 04 10 00 00 400 20 -11

2015 05 06 14 00 430 16 -8

2015 05 10 16 00 390 14 -11

2015 06 07 03 00 300 12 -4

2015 07 13 00 00 450 10 -5

2015 08 16 02 00 500 12 -6

2015 08 26 04 00 420 10 -10

2015 08 27 02 00 350 14 -12

2015 09 08 08 00 550 25 -8

2015 10 07 04 00 450 18 -8

2015 10 25 16 00 400 10 -2

2015 11 07 06 00 570 20 -10

2015 12 20 04 00 400 20 -17

2015 12 31 17 00 430 17 -15

2016 01 05 10 00 480 17 0

2016 01 19 12 00 450 17 -12

2016 02 02 18 00 360 10 -8

2016 03 06 08 00 450 18 -6

2016 04 14 08 00 400 09 -8

2016 06 23 05 00 350 09 0

2016 07 20 08 00 570 14 -5

2016 09 14 21 00 300 09 -7

2016 09 19 03 00 400 18 -10

2016 10 13 04 00 400 23 -17

2016 11 10 00 00 350 12 -10

2017 05 27 21 00 400 22 -21

2017 07 17 00 00 600 12 -6

——————————————————
